# Supplementary material for: The causal role of circulating inflammatory markers in osteoporosis: a bidirectional Mendelian randomized study
Source: Front Immunol. 2024 Jul 18;15:1412298. doi: 10.3389/fimmu.2024.1412298 (PMC11291241; doi:10.3389/fimmu.2024.1412298)
Supplement: Supplementary file 1 [file DataSheet_1.zip › supplementary material/CIM-OSTEOPOROSIS /GCST90274848.csv_forest.pdf]

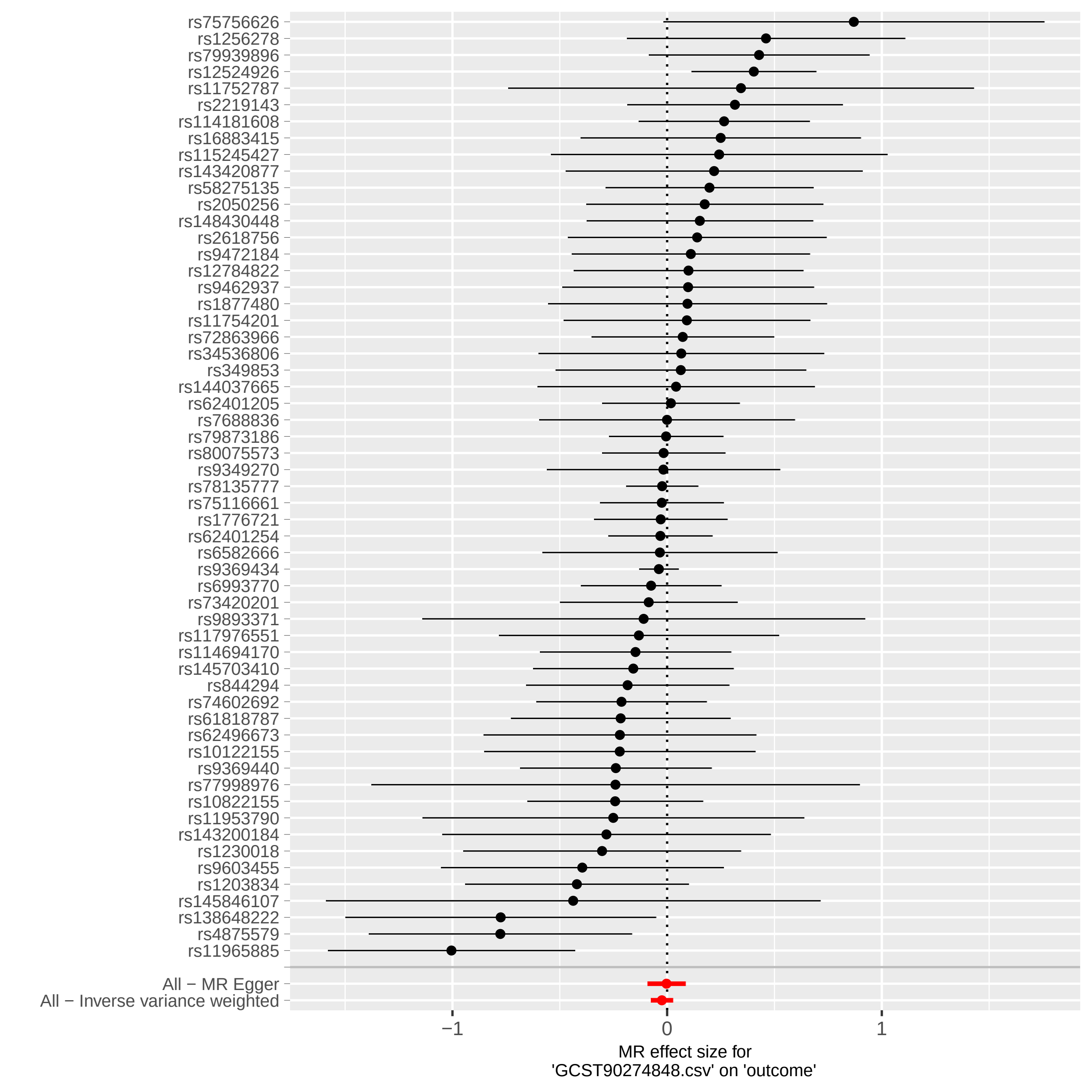
rs75756626
rs1256278
rs79939896
rs12524926
rs11752787
rs2219143
rs114181608
rs16883415
rs115245427
rs143420877
rs58275135
rs2050256
rs148430448
rs2618756
rs9472184
rs12784822
rs9462937
rs1877480
rs11754201
rs72863966
rs34536806
rs349853
rs144037665
rs62401205
rs7688836
rs79873186
rs80075573
rs9349270
rs78135777
rs75116661
rs1776721
rs62401254
rs6582666
rs9369434
rs6993770
rs73420201
rs9893371
rs117976551
rs114694170
rs145703410
rs844294
rs74602692
rs61818787
rs62496673
rs10122155
rs9369440
rs77998976
rs10822155
rs11953790
rs143200184
rs1230018
rs9603455
rs1203834
rs145846107
rs138648222
rs4875579
rs11965885
All − MR Egger
All − Inverse variance weighted
−1
0
1
MR effect size for
'GCST90274848.csv' on 'outcome'
